# Pronounced genetic differentiation in *Fokienia hodginsii* revealed by simple sequence repeat markers

**DOI:** 10.1002/ece3.4560

**Published:** 2018-10-12

**Authors:** Qianyi Yin, Sufang Chen, Wei Guo, Yanshuang Huang, Yelin Huang, Renchao Zhou, Qiang Fan, Wenbo Liao

**Affiliations:** ^1^ State Key Laboratory of Biocontrol and Guangdong Provincial Key Laboratory of Plant Resources Sun Yat‐sen University Guangzhou China; ^2^ Department of Horticulture and Landscape Architecture Zhongkai University of Agriculture and Engineering Guangzhou China

**Keywords:** conservation, endangered species, *Fokienia hodginsii*, genetic differentiation, microsatellite, southern China

## Abstract

*Fokienia hodginsii* is a Tertiary relict conifer of the monotypic genus *Fokienia* (Cupressaceae *s.l*.). Currently, the species is distributed in southern China, northern Vietnam, and northern Laos and listed as a “near threatened” species by the IUCN. In this study, a total of 427 individuals of *F. hodginsii* were sampled from China and Vietnam to characterize its genetic diversity and population differentiation. Based on the profiles of 12 simple sequence repeat (SSR) markers, we observed a high level of genetic diversity in *F. hodginsii* at the species level (*H*_e_ =0.635), albeit slightly lower than that of its sister species *Chamaecyparis obtusa*. Signals of bottleneck events were detected in the populations GXDMS, GXHJ, V‐PXB, and V‐HB, probably due to Pleistocene glaciations or overexploitation in recent years. Pronounced genetic differentiation (*F*_st_ *__*= 0.157) was found in this species. The inbreeding index (*F*_is_ *__= *0.176 ± 0.024) indicated that *F. hodginsii* has a mixed mating system. Significant correlation was found between the pairwise genetic differentiation and geographic distance (*r* = 0.882, *p* = 0.01), suggesting that genetic differentiation among the populations follows the model of isolation by distance (IBD). STRUCTURE analysis and principal coordinate analysis revealed that these populations were divided into four groups: the western China group located mainly in the Yunnan–Guizhou Plateau, the central China group located mostly in the Luoxiao Mountains and Nanling Mountains, the eastern China group located in the Wuyi Mountains and the Vietnam group containing two populations in Vietnam. The different terrains and elevations of populations may be the most likely factors leading to the differentiation between the western China group and the central China group, while the geographic isolation caused by the lack of appropriate habitats may greatly contribute to the differentiation between the central China group and the eastern China group. Based on the results, some conservation suggestions for this species are provided, such as establishing seed orchards and multiple nature reserves.

## 1 INTRODUCTION

Under current rapid global climate change, many endemic species are facing a high risk of extinction due to limited natural ranges resulting from genetic stochasticity or demographic, environmental, or other factors (Caughley, 1994; Gitzendanner & Soltis, 2000; Lande, 1993). It is vital to understand the genetic characteristics of these species, such as genetic diversity and population structure, for their management and the development of effective conservation strategies (Eckert, Samis, & Lougheed, 2008; Lesica & Allendorf, 2010).

The gymnosperm family Cupressaceae Bartling comprises approximately 22 genera and 150 species. Most of these species are Tertiary relict species that arose in the Jurassic (possibly as early as the Triassic), thrived in the Jurassic, and decreased in members continuously up to the present. It is also the only family of gymnosperms that is present on all continents except Antarctica (Yang, Ran, & Wang, 2012). However, except for *Juniperus*,* Sabina*, and *Cupressus*, most species in this family are locally endemic, and ensuring their survival under future climate change will require public and scientific attention.

**Figure 1:**
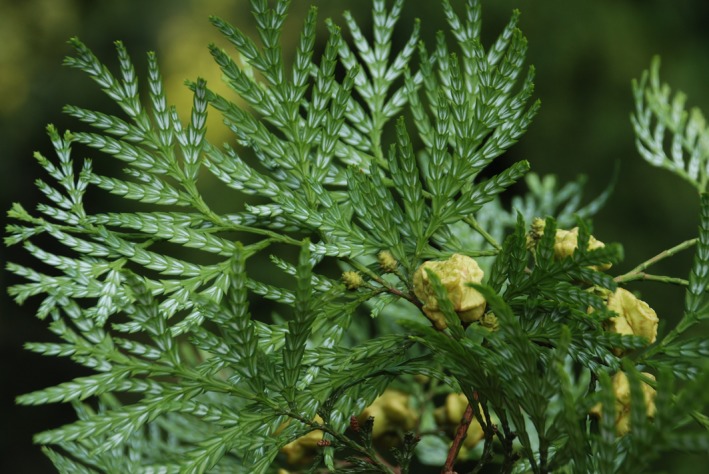
Photograph of *Fokienia hodginsii*

The genus *Fokienia* Henry et Thomas (Cupressaceae *s.l*.) contains only one extant species, *Fokienia hodginsii* (Dunn) Henry et Thomas (Farjon, 2005; Figure 1). Fossil records show that *Fokienia* was widely distributed in the Northern Hemisphere in ancient periods: fossils in forms with foliage and attached seed cones of *Fokienia* were reported from the Paleocene in Saskatchewan, central Canada (McIver & Basinger, 1990); the Oligocene in Jilin, northeastern China (Guo & Zhang, 2002); and the Miocene in Zhejiang, eastern China (He, Sun, & Liu, 2012). However, this genus is currently distributed in only southern China, northern Vietnam, and northern Laos (Zheng & Fu, 1978). In China, it occurs at elevations between approximately 1,000 and 1,800 m as a minor constituent of the subtropical evergreen (mixed) forest (Zheng & Fu, 1978). This conifer is a good landscape tree species with a beautiful shape and straight trunk (Huang et al., 2013) and is commonly cut down for building materials because of its light texture and material stability (Huang, Huang, Guo, & Zheng, 2015). Currently, this conifer is listed as “near threatened (NT)” as part of the International Union for Conservation of Nature Red List (IUCN 2004) and the National Secondary Protected Plants by Order of the Forestry Bureau and Ministry of Agriculture of China (https://www.gov.cn/gongbao/content/2000/content_60072.htm), the vulnerable species by the Information System of Chinese Rare and Endangered Plants (https://rep.iplant.cn/protlist), National Secondary Protected Plants in China and a K‐class protected plant species in Vietnam (Vuong, 2009).

Most recent studies on *F. hodginsii* mainly focused on seed breeding, nursery technology, plantation cultivation, essential oil extraction and development and utilization of other resources (Huang et al., 2013; Zhao, 2005). Only one paper mentioned the progress in genetics of *F. hodginsii*, according to Tam, Trang, and Hoa (2011), who investigated the genetic diversity and population structure of *F. hodginsii* in Vietnam by applying ISSR markers and showed that *F. hodginsii* maintained a low level of genetic variability and a high level of genetic differentiation. They supposed that human disturbance may play a key role in the present status of *F. hodginsii* by leading to the degradation and fragmentation of its habitats.

Simple sequence repeat (SSR; microsatellite) markers, codominant markers with good reproducibility and high variability, are one of the best tools to understand species genetic diversity and population structure (Wang, Huang, & Long, 2013). Based on transcriptome sequencing, we synthesized 108 SSR primers that were successfully amplified in *F. hodginsii* (Ding et al., 2017). Applying these SSR markers, we aimed to investigate the levels of genetic diversity and population structure of this species, which could provide some reliable information for the protection of this endangered species.

## 2 MATERIALS AND METHODS

### 2.1 Sample collection and DNA extraction

**Figure 2:**
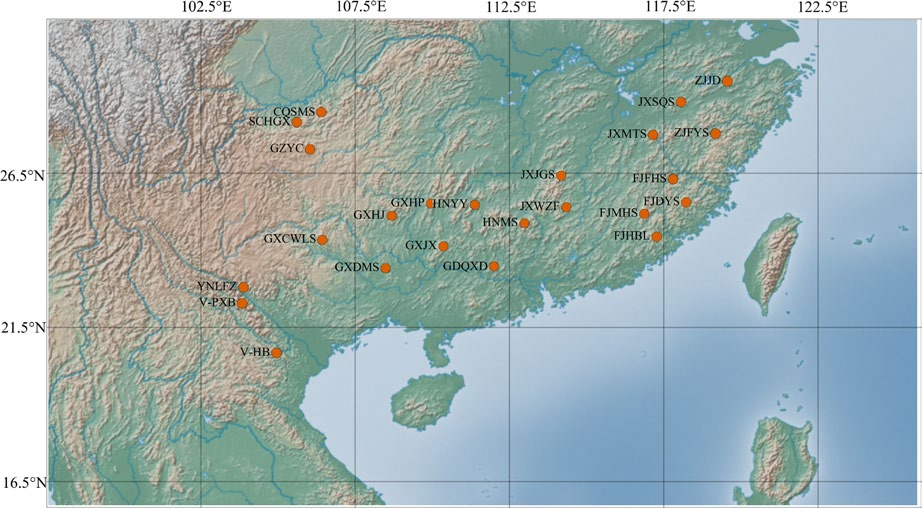
Geographic locations of the 24 populations of *Fokienia hodginsii*

A total of 427 individuals of *F. hodginsii* were sampled from 24 locations across twelve provinces of China and Vietnam (Table 1; Figure 2). A Garmin GPS unit (GPSMAP 62sc, Taiwan) was used to record the sample geographic locations with a margin of 10 m. For each population, fresh leaves were collected from 5 to 23 randomly selected fully grown individuals, which were at least 30 m apart from each other. Then, the leaf tissues were dried by silica gel and stored in zip‐lock plastic bags for DNA extraction. Voucher specimens for each population were all deposited in the Herbarium of Sun Yat‐sen University (SYS).

**Table 1:** Groups based on the result from SAMOVA and geographic information for populations of *Fokienia hodginsii*

Pop. ID	Geographic locality	Geographic coordinates	Altitude (m)	Sample size
The eastern China group				
ZJJD	Jiande, Zhejiang, China	119°33′19.98″E, 29°34′40.56″N	877	20
ZJFYS	Longquan, Zhejiang, China	119°10′11.05″E, 27°52′49.63″N	1,471	20
FJHBL	Nanjing, Fujian, China	117°15′38.83″E, 24°31′13.57″N	762	15
FJDYS	Dehua, Fujian, China	118°13′2.34″E, 25°38′27.1″N	1,095	20
FJFHS	Shaxian, Fujian, China	117°47′29.86″E, 26°23′32.6″N	369	20
FJMHS	Longyan, Fujian, China	116°51′17.78″E, 25°16′0.61″N	830	20
JXSQS	Shangrao, Jiangxi, China	118°3′50″E, 28°54′10.5″N	1,354	20
JXMTS	Zixi, Jiangxi, China	117°8′11.81″E, 27°50′6.31″N	805	11
The central China group				
GDQXD	Zhaoqing, Guangdong, China	111°57′56.82″E, 23°33′29.25″N	1,068	20
JXJGS	Jinggangshan, Jiangxi, China	114°09′16.36″E, 26°30′32.82″N	1,311	20
JXWZF	Shangyou, Jiangxi, China	114°19′12″E, 25°28′47.99″N	1,488	20
HNMS	Yizhang, Hunan, China	112°57′19.63″E, 24°57′49.43″N	1,103	20
HNYY	Daoxian, Hunan, China	111°20′45.39″E, 25°33′38.92″N	1,247	23
The western China group				
GXCWLS	Baise, Guangxi, China	106°22′36.07″E, 24°25′9.19″N	1671	20
GXDMS	Nanning, Guangxi, China	108°26′17.47″E, 23°29′46.39″N	1,203	5
GXHP	Longsheng, Guangxi, China	109°54′51.55″E, 25°36′14.52″N	1,290	20
GXHJ	Dongxing, Guangxi, China	108°38′23.94″E, 25°12′9.82″N	1,139	7
GXJX	Jinxiu, Guangxi, China	110°19′15.11″E, 24°12′40.19″N	989	20
YNLFZ	Mengzi, Yunnan, China	103°49′6.11″E, 22°52′12.27″N	1503	19
GZYC	Yuchong, Guizhou, China	105°58′50.32″E, 27°22′2.01″N	1,323	20
CQSMS	Jiangjin, Chongqing, China	106°20′55.27″E, 28°34′38.61″N	1,170	20
SCHGX	Xuyong, Sichuan, China	105°33′7.84″E, 28°14′40.64″N	1,122	20
The Vietnam group				
V‐PXB	Fansipan, Sapa, Vietnam	103°46′22.34″E,22°21′03.54″N	1823	11
V‐HB	Mai Châu, Hòa Binh, Vietnam	104°53′25.10″E,20°44′19.48″N	1,366	16

Total DNA was extracted from dried leaf tissue using the modified CTAB method (Doyle & Doyle, 1987). For each population, two individuals were randomly selected for PCR amplifications with all 108 primers designed by Ding et al. (2017). Fluorescence was added to the 3′ end of the 12 SSR markers (Table 2) with the highest polymorphism levels, and PCR amplifications were performed for all 427 individuals, in which the annealing temperature for each primer was set to 52°C. The PCR products were first inspected in 1% agarose gel and then electrophoresed on an Applied Biosystems 3730xl DNA Analyzer (Applied Biosystems, Foster City, California, USA).

**Table 2:** The information for the 12 microsatellites

Locus	Primer sequences (5′–3′)	Repeat	Expected size (bp)	Putative function
F015	F: TGTAATAACTCTGTCCCTTCC	(TA)7	200–210	Arabidopsis thaliana SIT4 phosphatase‐associated family protein
	R: CTCTGTGCTCCTCTCCAA			
F017	F: AAGACAAGATGCTCAGATCA	(AG)7	192–196	Picea glauca clone GQ03325_I06 mRNA
	R: GTGGTAGCCTAGAACTTCAT			
F020	F: TTCCTGCTTGAATGAATCCA	(CT)7	232–238	Arabidopsis thaliana armadillo/beta‐catenin repeat family protein
	R: GCGGAGGAGAAGGAGATT			
F036	F: GCCGAGACAGAGATAGAGA	(AG)6	260–268	Oryza sativa (japonica cultivar‐group) U1 small nuclear ribonucleoprotein 70 K
	R: ATAGCATAACAGCACCTCAT			
F042	F: TGGAAGAAGATATGGTCAAGG	(GA)6	264–270	Arabidopsis thaliana auxilin‐like protein
	R: TCAATAGCTGCTCTGTCAC			
F049	F: CAATGTTCCTTCTGTGTCTG	(CAG)7	221–245	Picea sitchensis clone WS02761_D24 unknown mRNA
	R: TTGATACTGAGGTGCTTGAA			
F089	F: TACGGATGAGCAGTCCAT	(TGG)5	276–291	Cryptomeria japonica putative glycine‐rich RNA binding protein
	R: CACCTCCACCACCATTAC			
F127	F: CCTTCAACTCATCATAGAATGG	(TTC)6	230–242	Not found
	R: TGAGCCTTCACTGCTAATG			
F173	F: TTATTCTACAGGCGAAGCAT	(AAC)5	194–206	Arabidopsis thaliana zinc‐binding family protein
	R: TATTCTGGATAAGACGGTGAG			
F204	F: TCTGGGAATGTTTGGGAAG	(CAG)5	201–210	Pisum sativum ultraviolet‐B‐repressible dehydrin‐related protein
	R: CTGCGTCTATAAAGCCTAATC			
F210	F: TGGAAGGAAGAAGGAAGATG	(GTG)5	291–306	Not found
	R: CGGACCTCATGTAAGAACTT			
F217	F: GCATATAAGGTGGCGACTC	(CAT)5	200–212	Pinus radiata PrLTP1
	R: GCAGGAAGTGGTGAGAAG			

### 2.2 Data analyses

Linkage disequilibrium (LD) between pairs of loci and deviation from Hardy–Weinberg equilibrium (HWE) for each locus/population combination were tested using ARLEQUIN version 3.1 (Schneider, Roessli, & Excoffier, 2000). Parameters of genetic variation were calculated using GenAlEx v6.41 (Peakall & Smouse, 2006), including the total number of alleles (*N*_a_), the effective number of alleles (*N*_e_), the expected and observed heterozygosities (*H*_e_ and *H*_o_, respectively), the Shannon information index (*I*) and the fixation (inbreeding) index (*F*_is_). Additionally, FSTAT version 2.9.3.2 (Goudet, 2002) was used to calculate the allelic richness (*A*_R_), the unbiased estimate of Wright's *F*‐statistic (including total‐population inbreeding coefficients (*F*_it_), the overall intrapopulation inbreeding coefficient (*F*_is_) and the interpopulation genetic differentiation coefficient (*F*_st_), Weir & Cockerham, 1984), and pairwise *F*_st_ between paired populations. Based on pairwise *F*_st_, gene flow between populations (*N*_m_) was further estimated with the following formula: *N*_m_ = (1 − *F*_st_)/4*F*_st_ (Wright, 1969). Four abiotic‐climate variables, namely, minimum temperature, maximum temperature, average temperature, and precipitation, from the sampled locations were obtained from the WorldClim database (Version 1.4; https://www.worldclim.org/) and used to calculate the differentiation matrix. Mantel tests (Mantel, 1967) between the matrix of the pairwise population differentiation in terms of *F*_st_/(1 − *F*_st_) and the differentiation matrix of geographic distances or abiotic‐climate variables were performed with GenAlEx with 1,000 random permutations (Rousset, 1997).

Taking into account the geographic location of each population and the genetic differentiation within and among populations, Spatial Analysis of Molecular Variance (SAMOVA) software (Dupanloup, Schneider, & Excoffier, 2002) was used to define the best number of groups; then, ARLEQUIN version 3.11 was used for the analysis of molecular variance (AMOVA; Excoffier, Smouse, & Quattro, 1992), in which three levels of genetic differentiation were calculated: genetic differentiation within populations, genetic differentiation among populations within groups, and genetic differentiation among groups.

BOTTLENECK 1.2.02 (Piry, Luikart, & Cornuet, 1999) was used to detect signals of recent bottleneck effects, in which one‐tailed Wilcoxon signed‐rank tests (10,000 replications) based on the “infinite allele model of mutation” (I.A.M.), the “stepwise mutation model’’ (S.M.M.), and the “two‐phased model of mutation” (T.P.M.; 70% of alleles under S.M.M.) were performed, and Bonferroni corrections for multiple tests were made.

In addition, a Bayesian clustering approach implemented in STRUCTURE v2.3.4 (Evanno, Regnaut, & Goudet, 2005) was used to investigate population structure, in which a 100,000 burn‐in period was followed by 10 iterations of 100,000 Markov chain Monte Carlo replicates per *K* (1–10). Then, STRUCTURE HARVESTER (Earl & Vonholdt, 2012) was used to determine the optimum *K*. Further, a principal coordinate analysis (PCoA) was conducted based on the Jaccard distance between populations using MVSP software (Kovach, 1999).

## 3 RESULTS

### 3.1 Genetic diversity

According to the LD analysis for these 12 polymorphic loci, no pairs of loci showed linkage disequilibrium after a sequential Bonferroni correction for multiple tests, indicating that the 12 markers can be considered independent markers for population genetics studies. The genetic variation across the 24 natural populations is summarized in Table 3. According to Table 3, a total of 78 alleles were detected from these 12 SSR loci, ranging from 4 to 8 per locus. The average allelic richness (*A*_R_) for each population ranged from 2.967 to 3.365 (average: 3.193 ± 0.067). The value of *N*_a_ ranged from 2.917 to 3.750 (average: 3.465 ± 0.044), *N*_e_ ranged from 2.461 to 3.106 (average: 2.861 ± 0.034), and *H*_e_ and *H*_o_ ranged from 0.551 to 0.669 (average: 0.635 ± 0.005) and 0.475 to 0.583 (average: 0.523 ± 0.007), respectively. After Bonferroni corrections, no loci showed deviations from Hardy–Weinberg equilibrium (Supporting Information Table S1). The *F*_is_ (inbreeding coefficient) averaged across all loci ranged from 0.048 to 0.326 (average: 0.176 ± 0.024, Table 4).

**Table 3:** Genetic variability for the 12 SSR markers within populations

Pop	*N*	*A*_R_	*N*_a_	*N*_e_	*H*_o_	*H*_e_	*F*_is_	*I*
ZJJD	42	3.181	3.5	2.877	0.533	0.639	0.161	1.117
ZJFYS	45	3.365	3.75	3.089	0.496	0.659	0.241	1.178
FJHBL	44	3.348	3.667	3.024	0.544	0.666	0.18	1.178
FJDYS	43	3.318	3.583	3.106	0.521	0.669	0.22	1.179
FJFHS	43	3.273	3.583	3.031	0.542	0.658	0.166	1.158
FJMHS	44	3.283	3.667	2.989	0.517	0.658	0.21	1.161
JXSQS	43	3.253	3.583	3.062	0.567	0.656	0.124	1.152
JXMTS	39	3.078	3.25	2.804	0.583	0.628	0.062	1.069
GDQXD	41	3.2	3.417	2.956	0.517	0.637	0.172	1.114
JXJGS	40	3.083	3.333	2.775	0.563	0.624	0.09	1.077
JXWZF	41	3.114	3.417	2.804	0.521	0.63	0.175	1.091
HNMS	42	3.251	3.5	3.023	0.563	0.662	0.147	1.156
HNYY	42	3.147	3.5	2.826	0.496	0.634	0.219	1.106
GXCWLS	44	3.15	3.667	2.624	0.496	0.604	0.174	1.076
GXDMS	39	3.25	3.25	2.517	0.533	0.59	0.286	1.011
GXHP	43	3.201	3.583	2.775	0.496	0.633	0.22	1.112
GXHJ	39	3.147	3.25	2.662	0.524	0.606	0.262	1.04
GXJX	44	3.244	3.667	3.048	0.475	0.661	0.084	1.159
YNLFZ	44	3.217	3.667	2.908	0.518	0.65	0.207	1.139
GZYC	42	3.2	3.5	2.923	0.479	0.651	0.129	1.134
CQSMS	41	3.135	3.417	2.87	0.488	0.645	0.242	1.109
SCHGX	42	3.244	3.5	3.034	0.542	0.662	0.181	1.155
V‐PXB	32	2.967	3	2.47	0.508	0.573	0.111	0.93
V‐HB	34	2.988	2.917	2.461	0.51	0.551	0.066	0.908
Mean		3.193 ± 0.067	3.465 ± 0.044	2.861 ± 0.034	0.522 ± 0.007	0.635 ± 0.005	0.172 ± 0.011	1.105 ± 0.012

Notes

*A*_R_: allelic richness; *F*_is_: coefficient of inbreeding; *H*_e_: expected frequency of heterozygotes; *H*_o_: observed frequency of heterozygotes; *I*: Shannon index; *N*: number of alleles; *N*_a_: observed number of alleles; *N*_e_: effective number of alleles.

**Table 4:** Genetic diversity at the 12 microsatellite loci

Loci	*N_T_*	*A*_R_	*N*_a_	*N*_e_	*H*_o_	*H*_e_	*F*_is_	*F*_it_	*F*_st_	*N*_m_
F015	8	4.233	4.000	3.405	0.583	0.700	0.167	0.284	0.140	1.533
F017	6	2.769	2.958	2.609	0.541	0.607	0.109	0.227	0.132	1.647
F020	5	4.071	3.250	2.846	0.429	0.636	0.326	0.411	0.126	1.730
F036	9	3.323	4.292	3.294	0.522	0.688	0.241	0.342	0.133	1.634
F042	4	3.520	3.917	3.213	0.552	0.686	0.195	0.216	0.025	9.610
F049	7	3.214	2.875	2.415	0.546	0.574	0.048	0.334	0.300	0.582
F089	5	3.339	3.000	2.415	0.531	0.568	0.065	0.250	0.198	1.012
F127	7	4.546	4.375	3.433	0.574	0.699	0.178	0.283	0.127	1.712
F173	7	3.926	3.125	2.452	0.407	0.589	0.308	0.431	0.178	1.158
F204	6	3.279	2.958	2.637	0.518	0.616	0.158	0.304	0.173	1.194
F210	8	3.947	3.625	2.721	0.520	0.617	0.156	0.323	0.198	1.016
F217	6	4.171	3.208	2.890	0.541	0.644	0.161	0.293	0.158	1.330
Mean		3.695 ± 0.044	3.465 ± 0.044	2.861 ± 0.034	0.522 ± 0.007	0.635 ± 0.005	0.176 ± 0.024	0.308 ± 0.019	0.157 ± 0.019	2.013 ± 0.698

*A*_R_: allelic richness, i.e. the average number of alleles per locus; *F*_is_: inbreeding coefficient; *F*_it_: total‐population inbreeding coefficient; *F*_st_: among‐population genetic differentiation coefficient; *H*_e_: unbiased expected heterozygosity; *H*_o_: observed heterozygosity; *N*_a_ observed number of alleles; *N*_e_: effective number of alleles; *N*_m_: gene flow; *N_T_*: number of alleles per locus.

Populations V‐PXB and V‐HB, located in Vietnam, had the lowest genetic diversity (V‐PXB: *H*_e_ = 0.573 and *H*_o_ **= 0.508; V‐HB: *H*_e_ **= 0.551 and *H*_o_ **= 0.510). Among the 22 populations in China, GXDMS and GXHJ harbored the lowest genetic diversity (*H*_e_ *__*= 0.590 and 0.606 and *H*_o_ **= 0.533 and 0.524, respectively). In contrast, the populations FJDYS, FJHBL, HNMS and SCHGX showed the highest genetic diversity (*H*_e_ = 0.662–0.669 and *H*_o_ = 0.521 – 0.563).

### 3.2 Genetic structure

**Figure 3:**
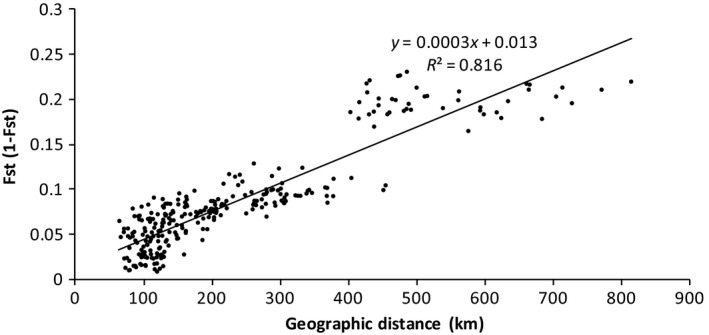
Relationship between pairwise *F*_st_/(1 − *F*_st_) and the geographic distance among the populations of *Fokienia hodginsii* (*r* = 0.882, *p* = 0.01)

The results from *F*‐statistics showed that the overall intrapopulation inbreeding coefficient (*F*_is_) was 0.176 ± 0.024, the total‐population inbreeding coefficient (*F*_it_) was 0.308 ± 0.019, the interpopulation genetic differentiation coefficient (*F*_st_) was 0.157 ± 0.019, and the gene flow (*N*_m_) was estimated to be 2.013 ± 0.698 (Table 4). All pairwise *F*_st_ values were highly significant (*p < *0.001), ranging from 0.009 (between FJDYS and FJFHS) to 0.234 (between V‐HB and ZJJD; Table 5). Correlation analyses showed that the genetic differentiation was most correlated with geographic distance (*r* = 0.882, *p = *0.01, Figure 3), longitudinal changes (*r* = 0.466, *p* = 0.01), latitudinal changes (*r* = 0.432, *p* = 0.01), precipitation differentiation (*r* = 0.256, *p* = 0.01), elevational changes (*r* = 0.205, *p* = 0.01), and average temperature changes (*r* = 0.178, *p* = 0.04; Table 6).

**Table 5:** Pairwise population matrix of gene flow (upper triangle) and *F*_st_ values (lower triangle) for all populations

POP	ZJJD	ZJFYS	FJHBL	FJDYS	FJFHS	FJMHS	JXMTS	JXSQS	JXJGS	JXWZF	GDQXD	HNMS	HNYY	GXJX	GXHP	GXHJ	GXDMS	GXCWLS	GZYC	CQSMS	SCHGX	YNLFZ	V‐PXB	V‐HB
ZJJD	0.000	15.728	16.894	8.984	7.049	9.803	8.197	4.429	2.827	2.753	3.140	3.343	2.949	2.733	2.423	1.929	2.384	2.214	2.446	2.433	2.446	2.272	1.028	0.834
ZJFYS	0.016	0.000	13.977	10.007	8.882	10.419	6.643	3.796	2.555	2.571	2.627	3.095	2.769	2.621	2.330	1.781	2.256	1.990	2.294	2.268	2.356	2.146	1.153	0.937
FJHBL	0.015	0.018	0.000	10.589	6.818	10.312	10.169	5.290	3.454	3.430	3.901	3.567	3.211	3.264	2.805	2.053	2.739	2.400	2.809	2.690	2.708	2.450	1.145	0.982
FJDYS	0.027	0.024	0.023	0.000	29.065	22.317	5.358	4.917	3.143	3.288	3.337	3.569	2.824	3.055	2.937	2.140	2.918	2.628	2.682	2.610	2.505	2.457	1.114	0.937
FJFHS	0.034	0.027	0.035	0.009	0.000	20.908	4.115	4.006	2.506	2.724	2.681	3.046	2.475	2.655	2.627	1.944	2.626	2.393	2.330	2.275	2.253	2.200	1.083	0.901
FJMHS	0.025	0.023	0.024	0.011	0.012	0.000	4.969	4.514	2.763	2.887	3.113	3.194	2.757	2.885	3.008	2.105	3.054	2.733	2.615	2.565	2.405	2.374	1.059	0.906
JXMTS	0.030	0.036	0.024	0.045	0.057	0.048	0.000	4.346	2.849	2.733	2.863	2.369	2.290	2.461	1.890	1.694	1.902	1.768	2.282	2.093	2.232	1.971	1.098	0.924
JXSQS	0.053	0.062	0.045	0.048	0.059	0.052	0.054	0.000	7.558	8.447	8.834	6.432	5.252	5.487	3.726	2.559	2.765	3.342	2.966	2.785	2.879	2.690	1.268	1.013
JXJGS	0.081	0.089	0.067	0.074	0.091	0.083	0.081	0.032	0.000	18.349	15.456	8.783	9.286	5.492	3.714	2.576	2.736	3.083	3.103	3.152	3.375	2.772	1.071	0.948
JXWZF	0.083	0.089	0.068	0.071	0.084	0.080	0.084	0.029	0.013	0.000	15.694	9.949	9.895	7.042	4.949	3.204	3.234	4.229	3.456	3.382	3.542	3.168	1.114	0.978
GDQXD	0.074	0.087	0.060	0.070	0.085	0.074	0.080	0.028	0.016	0.016	0.000	8.729	9.654	5.705	4.548	3.210	3.481	4.142	3.200	3.051	3.178	2.943	1.092	0.924
HNMS	0.070	0.075	0.066	0.065	0.076	0.073	0.095	0.037	0.028	0.025	0.028	0.000	16.444	8.039	7.419	3.663	4.156	4.843	4.550	4.413	4.601	4.243	1.224	1.078
HNYY	0.078	0.083	0.072	0.081	0.092	0.083	0.098	0.045	0.026	0.025	0.025	0.015	0.000	5.557	5.149	3.316	2.930	3.795	3.485	3.530	3.752	3.908	1.150	1.006
GXJX	0.084	0.087	0.071	0.076	0.086	0.080	0.092	0.044	0.044	0.034	0.042	0.030	0.043	0.000	11.990	4.966	6.925	8.235	8.724	7.952	9.582	5.730	1.096	0.995
GXHP	0.094	0.097	0.082	0.078	0.087	0.077	0.117	0.063	0.063	0.048	0.052	0.033	0.046	0.020	0.000	5.076	10.578	16.548	5.910	6.292	6.249	5.671	1.021	0.999
GXHJ	0.115	0.123	0.109	0.105	0.114	0.106	0.129	0.089	0.088	0.072	0.072	0.064	0.070	0.048	0.047	0.000	3.609	4.202	4.178	3.508	3.323	3.564	0.954	0.854
GXDMS	0.095	0.100	0.084	0.079	0.087	0.076	0.116	0.083	0.084	0.072	0.067	0.057	0.079	0.035	0.023	0.065	0.000	10.311	4.494	5.002	4.432	3.287	0.881	0.889
GXCWLS	0.101	0.112	0.094	0.087	0.095	0.084	0.124	0.070	0.075	0.056	0.057	0.049	0.062	0.029	0.015	0.056	0.024	0.000	4.992	5.596	4.800	4.649	0.899	0.857
GZYC	0.093	0.098	0.082	0.085	0.097	0.087	0.099	0.078	0.075	0.067	0.072	0.052	0.067	0.028	0.041	0.056	0.053	0.048	0.000	24.807	19.394	14.473	1.043	0.981
CQSMS	0.093	0.099	0.085	0.087	0.099	0.089	0.107	0.082	0.073	0.069	0.076	0.054	0.066	0.030	0.038	0.067	0.048	0.043	0.010	0.000	24.201	14.370	1.033	1.007
SCHGX	0.093	0.096	0.085	0.091	0.100	0.094	0.101	0.080	0.069	0.066	0.073	0.052	0.062	0.025	0.038	0.070	0.053	0.050	0.013	0.010	0.000	16.536	1.099	1.065
YNLFZ	0.099	0.104	0.093	0.092	0.102	0.095	0.113	0.085	0.083	0.073	0.078	0.056	0.060	0.042	0.042	0.066	0.071	0.051	0.017	0.017	0.015	0.000	1.115	1.087
V‐PXB	0.196	0.178	0.179	0.183	0.187	0.191	0.185	0.165	0.189	0.183	0.186	0.170	0.179	0.186	0.197	0.208	0.221	0.218	0.193	0.195	0.185	0.183	0.000	4.527
V‐HB	0.234	0.211	0.203	0.211	0.217	0.216	0.213	0.198	0.209	0.204	0.213	0.188	0.199	0.201	0.200	0.226	0.220	0.226	0.203	0.199	0.190	0.187	0.052	0.000

**Table 6:** The relationship between genetic differentiation (*F*_st_ /(1 ‐ *F*_st_)) and the differences in environmental factors

Influencing factors	Formula	*r*	*p*
Δ_min_ temperature	*y* = 0.0015*x* + 0.0808	0.067	0.27
Δ_average_ temperature	*y* = 0.0017*x* + 0.0798	0.178	0.04
Δ_max_ temperature	*y* = 0.0019*x *+ 0.0786	0.092	0.21
Δ precipitation	*y* = 4E−05*x* + 0.0676	0.256	0.01
Δ elevation	*y* = 3E−05*x *+ 0.00707	0.205	0.1
Δ latitude	*y* = 0.0094*x *+ 0.052	0.432	0.01
Δ longitude	*y* = 0.0043*x* + 0.0478	0.466	0.01

The SAMOVA demonstrated the highest value of *F*_CT_ (*F*_CT_ = 0.25346, *p* < 0.05; Supporting Information Figure S1) when it divided all 24 populations into four groups as follows: the western China group including the populations located in western China (mostly the Yunnan–Guizhou Plateau); the central China group including the populations located in central China (Luoxiao Mountains, Nanling Mountains, and adjacent areas); the eastern China group including the remaining populations, mostly in the Wuyi Mountains; and the last group including two populations in Vietnam. Based on this division, the AMOVA showed that genetic differentiation among groups accounted for 13.14% of the variation, genetic differentiation among populations within groups accounted for 2.20%, and genetic differentiation within populations accounted for 84.66% (Table 7). The gene flow among populations within groups and between different groups was also calculated. The results showed that the gene flow in the eastern China group had the maximum value (11.486) and that the Vietnam group had the minimum value (4.527) compared to the central China group (10.584) and the western China group (8.448). The gene flow between the eastern China group and the central China group was 2.960, and the gene flow between the western China group and the central China group was 3.892.

**Table 7:** Analysis of molecular variance (AMOVA) for the 24 populations

Source of variation	Sum of squares	Variance components	Percentage of variation	*F*‐statistics
Among groups	394.651	0.61683	13.14	*F*_st_:0.21430
Among populations within groups	169.975	0.10347	2.20	*F*_sc_:0.02538
Within populations	3277.343	3.97323	84.66	*F*_CT_:0.13142
Total	3841.969	4.69353	100.00	

**Figure 4:**
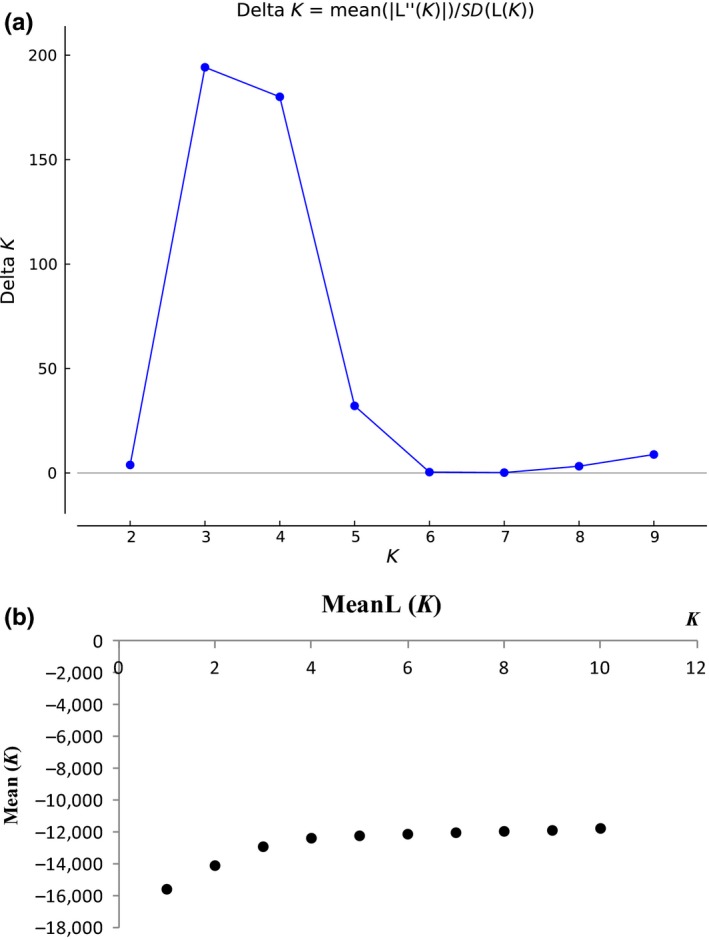
The best *K* value based on the result from STRUCTURE HARVESTER (a: Δ*K*; b: mean *L*(*k*))

**Figure 5:**
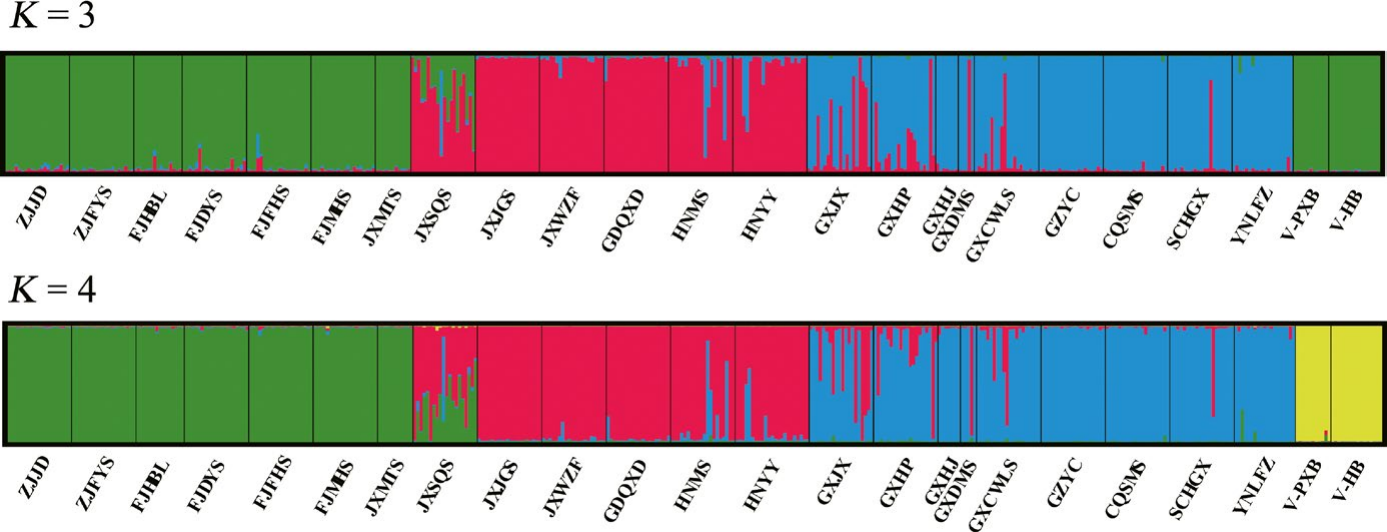
STRUCTURE individual assignment results for *K* = 3 and *K* = 4, based on simple sequence repeat data. Different colors represent different gene pools. *K* is the number of gene pools

**Figure 6:**
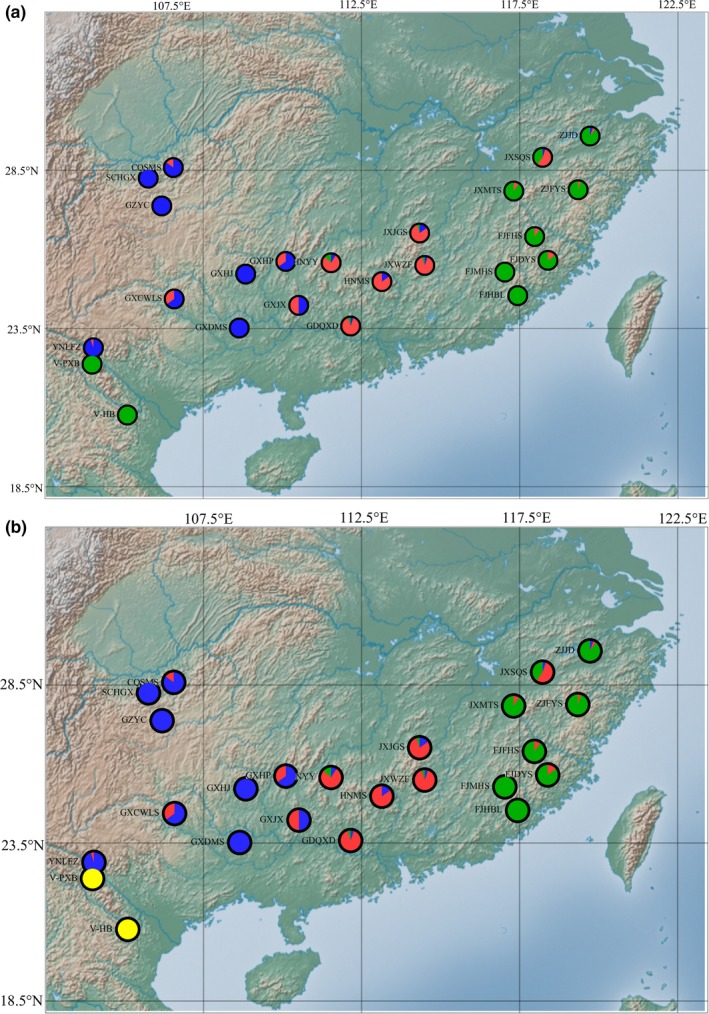
Grouping of populations according to STRUCTURE (*K* = 3 or *K* = 4) and their geographic locations

In the results of the STRUCTURE analysis, Δ*K* showed the highest value at *K* = 3 (Figure 4). Assignment results for *K* = 3 showed that all individuals could be roughly divided into three gene pools: the eastern China and Vietnam gene pool (mainly in green), the central China gene pool (mainly in red), and the western China gene pool (mainly in blue; Figure 5). When *K* = 4, the eastern China and Vietnam gene pool were divided into the eastern China gene pool (mainly in green) and the Vietnam gene pool (mainly in yellow; Figure 5), which agreed with the four groups divided by the SAMOVA (Figure 6).

**Figure 7:**
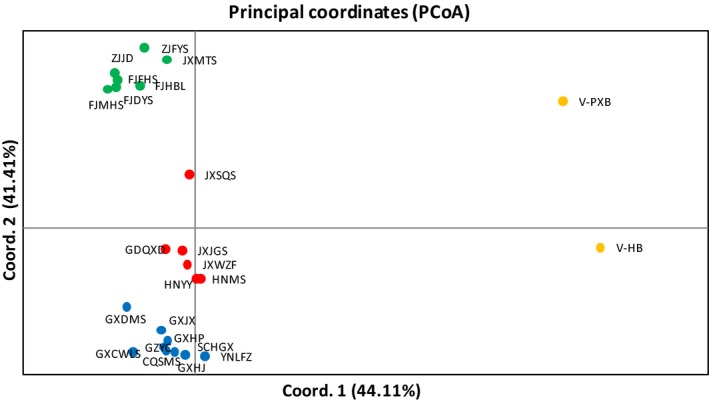
Principal coordinate analysis of individual genotypes obtained from four groups

Principal coordinate analysis showed that most populations of the western China group were located on the lower left side; populations of the central China group, on the middle left side; populations of the eastern China group, on the upper left side; and populations of the Vietnam group, on the right side (Figure 7).

### 3.3 Genetic bottleneck assessments

The Wilcoxon test and sign test indicated that bottleneck events may have occurred in the populations GXDMS, GXHJ, V‐PXB, and V‐HB via the infinite allele model and the two‐phased mutation model (Table 8).

**Table 8:** Results of bottleneck analyses for each population

POP ID	Wilcoxon test		Sign test		Model shift test
	I.A.M.	T.P.M.	I.A.M.	T.P.M.	
ZJJD	0.0744	0.1618	0.2645	0.0623	L‐shaped
ZJFYS	0.0853	0.1543	0.4768	0.1857	L‐shaped
FJHBL	0.1034	0.1764	0.3783	0.2879	L‐shaped
FJDYS	0.0953	0.1665	0.0624	0.2645	L‐shaped
FJFHS	0.0847	0.1555	0.1742	0.6829	L‐shaped
FJMHS	0.0963	0.1685	0.5305	0.1198	L‐shaped
JXSQS	0.0748	0.133	0.3195	0.0456	L‐shaped
JXMTS	0.0764	0.1319	0.381	0.2663	L‐shaped
GDQXD	0.0608	0.1338	0.5969	0.6244	L‐shaped
JXJGS	0.0608	0.1219	0.3142	0.2091	L‐shaped
JXWZF	0.0543	0.1256	0.3201	0.3694	L‐shaped
HNMS	0.0814	0.1706	0.3142	0.2377	L‐shaped
HNYY	0.0764	0.1391	0.12	0.1542	L‐shaped
GXCWLS	0.0975	0.625	0.1857	0.2645	L‐shaped
**GXDMS**	**0.0159**	**0.0312**	**0.0288**	**0.048**	L‐shaped
GXHP	0.1019	0.1497	0.6829	0.6238	L‐shaped
**GXHJ**	**0.0102**	**0.0096**	**0.0268**	**0.0379**	L‐shaped
GXJX	0.0858	0.1531	0.1238	0.1742	L‐shaped
YNLFZ	0.0921	0.16	0.4487	0.5305	L‐shaped
GZYC	0.0715	0.1479	0.2397	0.3192	L‐shaped
CQSMS	0.091	0.1624	0.0803	0.3711	L‐shaped
SCHGX	0.0784	0.1574	0.3169	0.4143	L‐shaped
**V‐PXB**	**0.0472**	**0.0264**	**0.0278**	**0.0326**	L‐shaped
**V‐HB**	**0.0376**	**0.0473**	**0.0154**	**0.0471**	L‐shaped

Note

I.A.M.: infinite allele model of mutation; T.P.M.: two‐phased model of mutation.

The bold values represent the significance values lower than 0.05 (*p* < 0.05).

## 4 DISCUSSION

### 4.1 Genetic diversity

Genetic diversity is crucial for species, as it may influence the ability of species to cope with environmental change (Frankham, Ballou, & Briscoe, 2002; Frankham, 1995a, 1995b). In this study, microsatellite markers were used to estimate population genetic diversity and to investigate the genetic structure of *F. hodginsii*. Slightly lower genetic diversity was found in *F. hodginsii* (*H*_e _= 0.635 ± 0.005) than in *Chamaecyparis obtusa* (*H*_e_ *__*= 0.780), the sister species of *F. hodginsii* (Matsumoto, Uchida, Taguchi, Tani, & Tsumura, 2010). Compared to other species (Nybom, 2004), the expected heterozygosities (*H*_e_) of *F. hodginsii* are similar to those of regional species (*H*_e_ *__*= 0.65) and long‐lived woody perennial species (*H*_e_ *__*= 0.68). Allelic diversity (*N*_a_) and expected heterozygosity (*H*_e_) are also commonly used to estimate the genetic diversity in natural populations (Freeland, Kirk, & Petersen, 2011; Hamilton, 2009). The *H*_e_ and *N*_a_ values of *F. hodginsii* (*H*_e_ = 0.635, *N*_a_ = 3.465) are slightly lower than those of *C. obtusa* (*H*_e_ = 0.780, *N*_a_ = 7.038), albeit higher than those of other conifer species, such as *Cryptomeria japonica* (*H*_e_ = 0.277, *N*_a_ = 2.000, Tsumura & Tomaru, 1999).

In this study, the lowest genetic diversity was found in the two populations in Vietnam (V‐PXB: *H*_e_ = 0.573; V‐HB: *H*_e_ = 0.551). This phenomenon agreed with previous reports that most populations in Vietnam harbor low genetic diversity (*H_T _*= 0.0970 ± 0.0101, ISSR markers used by Tam et al., 2011). It is possible that China serves as the central distributional area of *F. hodginsii*, such that its genetic diversity decreased as it dispersed from its central area to its marginal areas, such as Vietnam (Wei, Sork, Meng, & Jiang, 2016). Tam et al. (2011) also indicated that, as a result of human disturbance, the *F. hodginsii* habitat in Vietnam has been degraded and fragmented, which may also serve as a good explanation for the low genetic variability in Vietnam, as signals of bottleneck events were also detected in these two populations.

In China, the populations GXDMS and GXHJ, where only 5–7 individuals were collected, had the lowest genetic diversity (*H*_e_ *__*= 0.590 and 0.606, respectively), and signals of bottleneck events were also detected in these two populations (Table 8). These phenomena may be explained by insufficient sampling. However, as a Tertiary relict species, this conifer was strongly influenced by the Pleistocene glaciations, resulting in the populations contracting sharply. In China, it has been more than 2,600 years since this conifer was used to build boats and houses, and due to extensive deforestation, the lower distribution limit of this conifer has moved up by 500 m since the 1980s (Hou, Cheng, Lin, & Yu, 2004). During our field investigations, we also observed substantial evidence of deforestation near the *F. hodginsii* populations, and in many places where ample specimens were recorded, few or no individual were found, especially in the populations of GXDMS and GXHJ. Further, the geographic locations of these two populations were near Vietnam, indicating that the low genetic diversity observed in GXDMS and GXHJ may be caused by the same factors that account for the low genetic diversity observed in Vietnam.

### 4.2 Genetic differentiation

Most conifers have high levels of genetic diversity within populations and low levels of differentiation among populations (Hamrick, Godt, & Sherman‐Broyles, 1992). According to the AMOVA results in this study, the genetic diversity of *F. hodginsii* is primarily maintained within populations (84.66%, *p* < 0.01), while the genetic differentiation among populations of *F. hodginsii* (*F*_st_ *__= *0.157 ± 0.019) is weak; however, the value of *F*_is_ was 0.176 ± 0.024, indicating a mixed mating system in which inbreeding occurred frequently. The genetic differentiation among populations of *F. hodginsii* (*F*_st_ **= 0.157 ± 0.019) is also in accordance with that of other mixed‐breeding species of seed plants (79.2%, Nybom & Bartish, 2000), slightly higher than that of wind‐dispersed species (*F*_st_ *= *0.13), and much lower than that of entomophilous species (*F*_st_ **= 0.21) (Nybom, 2004). This pattern is also in accordance with previous observations that the dispersal of *Fokienia* is mainly through the wind, though sometimes also through insects (Jin et al., 2012; Lu et al., 2011; Wang & Ran, 2014). Such patterns were also observed in *Cupressus funebris*, for which the genetic diversity within populations is 88.15%, *F*_st_ = 0.1580 and *F*_is_ **= 0.1579 (Lu et al., 2014). For the species *C. obtusa*, much higher genetic diversity was maintained within populations (91.7%), and genetic differentiation among populations was lower (*F*_st_ = 0.039). The *F*_is_ value estimated for *C. obtusa* was only 0.034, indicating a random mating system. Therefore, the different levels of genetic differentiation among the three species may be caused primarily by the differentiation of mating systems.

In this study, a significant correlation was found between genetic differentiation (*F*_st_/(1 − *F*_st_)) and geographic distance (*r* = 0.882, *p* = 0.01), suggesting that the genetic differentiation among populations follows the model of isolation by distance (IBD), that is, the differentiation among populations is strongly associated with geographic distance. Such a phenomenon was also observed in *C. obtusa* (*r* ^2^ = 0.3997 and *p* = 0.001, Matsumoto et al., 2010). It is also known that the dispersal of *Fokienia* is mainly through the wind (Jin et al., 2012; Lu et al., 2011; Wang & Ran, 2014); thus, its capability for long‐distance dispersal could be limited as the geographic distance increases.

Although significant correlations were also found between genetic differentiation and climatic variables in the sampled locations, such as average temperature (*r* = 0.178, *p* = 0.04) and precipitation (*r* = 0.256, *p* = 0.01), their correlations were rather weak compared to those with geographic distance (*r* = 0.882, *p* = 0.01). It was observed that the flowering period of *F. hodginsii* is delayed with a decrease in temperature and precipitation (Hou et al., 2006); therefore, climatic factors may also actively increase the genetic differentiation among populations to a lesser extent.

### 4.3 Population structure

The STRUCTURE model based on 12 loci identified three as the most likely number of genetic clusters, as the highest Δ*K* value was at *K* = 3. The assignment results for *K* = 3 showed that the two populations in Vietnam were clustered with the eastern China group. In contrast, the results for *K* = 4 showed that the Vietnam populations were separated from the eastern China group and clustered as a fourth group. However, the populations located in Vietnam are located far away from those in eastern China, and the climatic conditions are much different between the two regions. It is surprising that the two populations in Vietnam were clustered with the eastern China group and not the western China group, which is much closer to Vietnam in terms of geographic distance. More molecular data need to be analyzed to understand this pattern.

In this study, the assignment results for *K* = 4 were the same as the results from SAMOVA and PCoA. Therefore, it is reasonable to divide all populations into four groups: the eastern China group, the central China group, the western China group, and the Vietnam group. The terrain of China from west to east forms a flight of three steps, commonly called the “Three Steps”. The first step located in southwestern China mainly includes the Qinghai‐Tibetan Plateau, which has an elevation above 4,000 m. The second step lies in central and western China with an elevation of 1,000–3,000 m and includes the Xuefeng Mountains, Qinling Mountains, and Yunnan–Guizhou Plateau. The third step spans all remaining regions, covering eastern and southern China with an elevation of 500 m (Huang et al., 2012). The western China group is located on the second step, which mainly contains plateau and basin, while the central China group and the eastern China group are located on the third step, which mainly contains plain and hills. Additionally, the elevation of the sampled populations in the western China group is generally higher than that of populations in the central China group and eastern China group (Table 1). According to Hou et al. (2006), the flowering period of *F. hodginsii* is delayed with an increase in elevation. Therefore, the change in topography may be the main reason for the population differentiation between the western China group and the central China group. Based on the specimen records and our field collections, the distribution of *F. hodginsii* is continuous between the western China group and the central China group; thus, populations located near the border, such as GXJX and GXHP, may receive gene flow from both groups and ultimately harbor mixed gene pools.

Population differentiation was also found between the central China group and the eastern China group even though both of them are located on the third step. It was found that the central China group belongs to the Guangdong and Guangxi Hills while the eastern China group belongs to the Zhejiang and Fujian Hills, and between them, most areas are plains with a low elevation where no specimen records of *F. hodginsii* were found. Therefore, the plain area between the central and eastern China groups may have limited the gene flow between them and led to genetic differentiation, as we have found that isolation by distance was the main reason for genetic differentiation of *F. hodginsii*. However, it was surprisingly that the population JXSQS, located in the eastern China group, was closer to the central China group genetically (Figure 5). It is possible that some of the individuals could be later generations of ancient transplants from the central area, considering that *F. hodginsii* was often planted around the tombs and temples in China.

### 4.4 Conservation implications

Genetic diversity plays an important role in determining the survival and adaptability of a species (Liao et al., 2015). The high genetic diversity maintained within *F. hodginsii* and the initial significant genetic differentiation among its populations found in this study are encouraging. However, we found recent bottleneck events in the populations GXDMS, GXHJ, V‐PXB, and V‐HB, suggesting that individual populations may suffer from a dramatic decline in population size. As a Tertiary relict species, the range of this conifer contracted sharply during the Pleistocene glaciations, and our field investigations also showed that the *F. hodginsii* populations have been overexploited since the 1980s, especially in the last ten years. For the conservation of this species, measures should be taken to increase the number of individuals and avoid the destruction caused by human activities. Ex situ conservation and breeding can also be considered to maintain the greatest within‐species genetic variation, especially for the populations GXHJ and GXDMS, with higher inbreeding coefficients. Establishing seed orchards is also a good method, which could preserve favorable genes and prepare for breeding in the future. According to the results from STRUCTURE, the optimum number of groups is 4; thus, we also should establish seed orchards for these four groups to preserve their genotypes. In addition, establishing multiple *F. hodginsii* nature reserves, such as the Daiyunshan National Nature Reserve and Nanling National Nature Reserve, is needed, and the communities containing *F. hodginsii* should be classified as absolute protection areas to avoid human destruction.

## CONFLICT OF INTEREST

None declared.

## AUTHORS’ CONTRIBUTIONS

Liao, W.B. and Fan, Q. designed the research. Guo, W. and Huang, Y.SH. collected the samples. Yin, Q.Y., Huang, Y.L. and Zhou, R.CH. generated the data. Yin, Q.Y., Chen, S.F. and Zhou, R.CH. analyzed and interpreted the data. Yin, Q.Y. wrote the manuscript, and Chen, S.F. and Zhou, R.CH. edited the manuscript.

## DATA ACCESSIBILITY

The primers used in this study are shown in Table 2, and all other data supporting the findings are available within the article and supplementary information file.
